# Association of Adherence to Healthy Lifestyle Recommendations With All-Cause and Cause-Specific Mortality Among Former Smokers

**DOI:** 10.1001/jamanetworkopen.2022.32778

**Published:** 2022-09-22

**Authors:** Maki Inoue-Choi, Yesenia Ramirez, Ami Fukunaga, Charles E. Matthews, Neal D. Freedman

**Affiliations:** 1 Metabolic Epidemiology Branch, Division of Cancer Epidemiology and Genetics, National Cancer Institute, Bethesda, Maryland; 2 Department of Epidemiology and Prevention, Center for Clinical Sciences, National Center for Global Health and Medicine, Tokyo, Japan

## Abstract

**Importance:**

The benefits of smoking cessation are well known, but former smokers have a higher health risk than never smokers. The impact of former smokers’ engaging in other aspects of a healthy lifestyle is unclear.

**Objective:**

To assess the association between adherence to evidence-based lifestyle recommendations and mortality among former smokers.

**Design, Setting, and Participants:**

This prospective cohort study included 159 937 participants in the National Institutes of Health–AARP Diet and Health Study of older US adults who completed the baseline and risk factor questionnaires and self-identified as former smokers. Baseline questionnaires were mailed from 1995 to 1996. Data analysis was performed from November 2020 to November 2021.

**Exposures:**

Adherence to evidence-based lifestyle recommendations was scored for body weight (scores, 0-2), diet (scores, 0-3), physical activity (scores, 0-2), and alcohol intake (scores, 0-1) recommendations, with higher scores indicating better adherence. Individual lifestyle adherence scores were summed to make a total adherence score (scores, 0-8).

**Main Outcomes and Measures:**

The primary outcomes were all-cause and cause-specific mortality through December 31, 2019, with a mean (SD) follow-up of 18.9 (6.3) years. Hazard ratios (HRs) and 95% CIs were computed using a multivariable Cox proportional hazards regression model.

**Results:**

Among 159 937 former smokers (mean [SD] age, 62.6 [5.2] years; 106 912 [66.9%] male; 149 742 [93.6%] White), 86 127 deaths occurred. A higher total adherence score was associated with lower all-cause mortality (HR per unit increase, 0.95; 95% CI, 0.94-0.95). Compared with the lowest total adherence score category (scores, 0-2), the HRs for all-cause mortality were 0.88 (95% CI, 0.86-0.90) for scores of 3 to 4, 0.80 (95% CI, 0.79-0.82) for scores of 5 to 6, and 0.73 (95% CI, 0.71-0.75) for scores of 7 to 8. Associations were observed regardless of health status, comorbid conditions, the number of cigarettes participants used to smoke per day, years since cessation, and age at smoking initiation. When examined individually, the HRs for highest vs lowest adherence score were 0.86 (95% CI, 0.84-0.88) for body weight, 0.91 (95% CI, 0.90-0.93) for diet, 0.83 (95% CI, 0.81-0.85) for physical activity, and 0.96 (95% CI, 0.94-0.97) for alcohol intake recommendations. Participants with a higher total adherence score also had a lower risk of mortality from cancer, cardiovascular disease, and respiratory disease.

**Conclusions and Relevance:**

In a large US cohort of former smokers, better adherence to healthy lifestyle recommendations was associated with lower mortality risk. These results provide evidence that former smokers may benefit from adhering to lifestyle recommendations, as do other groups.

## Key Points

**Question:**

Are healthy lifestyle choices associated with reduced mortality among former smokers?

**Findings:**

In this cohort study of 159 937 former smokers in the National Institutes of Health–AARP Diet and Health Study, former smokers who reported the highest adherence to recommendations for body weight, diet, physical activity, and alcohol intake had a 27% lower risk of all-cause mortality compared with those who reported the lowest adherence. Associations were also observed for deaths from cancer, cardiovascular disease, and respiratory disease.

**Meaning:**

These findings suggest that former smokers may additionally lower their risk of premature death by adhering to healthy lifestyle recommendations.

## Teaser

This cohort study of former smokers assesses whether adherence to healthy lifestyle recommendations is associated with reduced mortality.

## Introduction

Cigarette smoking is the leading cause of preventable disease and death in the US and worldwide.^1,2^ Tobacco use causes 480 000 deaths in the US and more than 8 million deaths worldwide each year.^1,3,4^ However, increasing awareness of the harms of smoking and concerted tobacco control policies have led to substantial rates of smoking cessation, reducing the prevalence of cigarette smoking by approximately two-thirds and averting millions of premature deaths.^1,5^ Currently, the number of former smokers in the US population (52.1 million) is higher than the number of current smokers (34.2 million).^1,6,7^

Extensive evidence supports health benefits of smoking cessation, including reduced risk of cancer, cardiovascular disease (CVD), and chronic respiratory disease, and increased longevity.^7,8^ Nevertheless, former smokers largely remain at higher mortality risk than never smokers and may additionally benefit from other aspects of a healthy lifestyle.^9,10,11^ Evidence-based lifestyle recommendations, such as lifestyle guidelines by the World Cancer Research Foundation (WCRF)/American Institute for Cancer Research (AICR) and American Cancer Society (ACS) for cancer prevention and by the American Heart Association/American College of Cardiology for CVD prevention, include maintaining a healthy body weight, being physically active, eating a healthy diet, and limiting alcohol consumption in addition to avoiding smoking.^12,13,14^ Better adherence to those lifestyle guidelines has been associated with lower disease and mortality risk in a number of previous studies, including meta-analyses.^15,16,17,18,19^ However, studies focused on former smokers have been limited, with most studies including cessation as 1 of the recommendations or adjusting the analysis for smoking. Thus, despite substantial potential benefits, it remains unclear whether former smokers also benefit from adhering to other healthy lifestyle recommendations. In this study, we assessed the association between adherence to evidence-based lifestyle recommendations and all-cause and cause-specific mortality among 159 937 former smokers in the US.

## Methods

### Study Population

Our study population was selected from the National Institutes of Health–AARP (NIH-AARP) Diet and Health Study, a large prospective cohort study of US adults.^20^ From 1995 to 1996, a baseline questionnaire was mailed to 3.5 million AARP members aged 50 to 69 years who resided in 1 of 6 states (California, Florida, Pennsylvania, New Jersey, North Carolina, and Louisiana) or 2 metropolitan areas (Atlanta, Georgia, and Detroit, Michigan), and 567 169 persons completed the questionnaire. This study was approved by the Special Studies Institutional Review Board of the National Cancer Institute. Participants were considered to have provided informed consent by completing and returning the baseline questionnaire. This study followed the Strengthening the Reporting of Observational Studies in Epidemiology (STROBE) reporting guideline.

### Exposure Assessment

A baseline questionnaire assessed demographic, anthropometric, medical, smoking, and other lifestyle factors, including physical activity and diet. Participants who self-identified as former smokers were asked to report the number of cigarettes per day (CPDs) they used to smoke and how long ago they had quit smoking. Body mass index (BMI) was calculated using self-reported weight and height as weight in kilograms divided by height in meters squared. Dietary intake during the past 12 months was assessed using an early version of the National Cancer Institute Dietary History Questionnaire, asking the frequency of consumption and corresponding portion sizes of 124 food items.^21^ Physical activity was assessed in detail on the risk factor questionnaire that was mailed to participants 6 to 12 months after the baseline questionnaire. Participants reported how often they participated in moderate and vigorous activities (eg, biking, fast walking, dancing, jogging, running, aerobics, hiking, swimming, heavy gardening, tennis, or heavy lifting). Detailed lifetime smoking, including age at which the participant started smoking regularly, was additionally collected on the 2004-2005 follow-up questionnaire.

Adherence score was calculated for body weight, diet, physical activity, and alcohol consumption recommendations using the scoring system outlined in Table 1. For body weight, we scored participants based on their BMI using the World Health Organization’s classifications: obese or underweight, 30 or higher or less than 18.5 (score, 0; no adherence); overweight, 25 to less than 30 (score, 1; partial adherence); and normal, 18.5 to less than 25 (score, 2; full adherence). For the dietary intake quality measure, we used the Healthy Eating Index 2015, which assesses how well a set of foods aligns with key recommendations of the *Dietary Guidelines for Americans, 2010-2015* and contains 13 components that sum to the maximum score of 100 points.^22^ The adherence score was assigned by Healthy Eating Index 2015 quartiles from the lowest (score, 0) to the highest (score, 3).^3^ Physical activity was evaluated based on the *Physical Activity Guidelines for Americans*, second edition.^23,24^ Participants were scored based on their reported time of moderate or vigorous physical activity: never or rarely (score, 0; no adherence), less than 1 to 3 hours per week (score, 1; partial adherence), and 4 hours per week or more (score, 2; full adherence). The adherence score for alcohol intake was based on the alcohol consumption recommendation by the *Dietary Guidelines for Americans, 2020-2025*.^25^ Women who reported drinking 1 or fewer alcoholic drinks per day and men who reported drinking 2 or fewer alcoholic drinks per day were scored as adherent (score, 1). Participants who reported drinking more than these amounts received an adherence score of 0. Scores for each of the individual recommendations were then summed to a total adherence score (range, 0-8 points), with higher scores indicating a higher level of adherence to healthy lifestyle parameters. The distributions of individual lifestyle recommendation adherence scores by total adherence score are given in eTable 1 in the Supplement.

**Table 1.** Scoring System of Adherence to the Body Weight, Diet, Alcohol Intake, and Physical Activity Recommendations

Score^a^	Definition	No. (%) of study participants
Body weight (BMI)		
2	18.5 to <25 (normal)	50 026 (31.3)
1	25 to <30 (overweight)	70 400 (44.0)
0	<18.5 or ≥30 (underweight or obese)	39 511 (24.7)
Diet (Healthy Eating Index 2015 total score)		
3	Quartile 4 (range, 75.2-95.7)	39 986 (25.0)
2	Quartile 3 (range, 69.3-75.2)	39 985 (25.0)
1	Quartile 2 (range, 62.5-69.3)	39 982 (25.0)
0	Quartile 1 (range, 24.0-62.5)	39 984 (25.0)
Physical activity (moderate or vigorous physical activity)		
2	≥4 h/wk	80 812 (50.5)
1	<1-3 h/wk	56 604 (35.4)
0	Never or rarely	22 521 (14.1)
Alcoholic beverage consumption		
1	≤2 drinks/d (men); ≤1 drink/d (women)	132 048 (82.6)
0	>2 drinks/d (men); >1 drink/d (women)	27 889 (17.4)

Abbreviation: BMI, body mass index (calculated as weight in kilograms divided by height in meters squared).

^a^ Higher scores indicate better adherence.

### Follow-up and Outcome Ascertainment

Among 327 946 individuals who completed the baseline questionnaire and the risk factor questionnaire, 163 781 (49.9%) self-identified as former smokers. We excluded 1660 individuals (1.0%) whose risk factor questionnaire was completed by a proxy and 2157 individuals (1.3%) with incomplete physical activity data, resulting in 159 937 former smokers (97.7%). Participants were followed up from the date when the returned baseline questionnaires were scanned until death or December 31, 2019, whichever occurred first.

Vital status and causes of death were ascertained by annual linkage of cohort participants to the Social Security Administration Death Master File, supplemented by the National Death Index, with a nearly complete follow-up. Cause-specific mortalities were defined using *International Classification of Diseases, Ninth Revision* (*ICD-9*) and *International Statistical Classification of Diseases and Related Health Problems, Tenth Revision* (*ICD-10*) codes as follows: all cancer (*ICD-9*: 140-208, 238.6; *ICD-10*: C00-C96), CVD (*ICD-9*: 390-398, 401-404, 410-429, 430-438, 440-448; *ICD-10*: I05-I16, I20-I52, I60-69, I70-I79), and respiratory disease (pneumonia, influenza, chronic lower respiratory disease; *ICD-9*: 480-487, 490-496; *ICD-10*: J09-J18, J40-J47).

### Statistical Analysis

Data analysis was performed from November 2020 to November 2021. We created 4 categories of the total adherence score: 0 to 2 (10.3%), 3 to 4 (33.5%), 5 to 6 (38.9%), and 7 to 8 (17.3%) based on the distribution. We estimated hazard ratios (HRs) and 95% CIs for all-cause and cause-specific mortality using Cox proportional hazards regression models with person-years as the underlying time metric. The lowest adherence score category was the referent group for all analyses. Covariates were determined by literature review and included age, sex, race and ethnicity (Hispanic, non-Hispanic Black, non-Hispanic White, or other [Asian, Pacific Islander, or American Indian/Alaskan Native]), educational level (high school or less, post–high school training, some college, or college or graduate school), perceived general health (excellent or very good, good or fair, or poor), time since quitting smoking (<1, 1-4, 5-9, or ≥10 years), and smoking intensity (1-10, 11-20, 21-30, 31-40, 41-60, or >60 CPDs). Race and ethnicity were included because the adherence to lifestyle recommendations and its association with mortality could differ by race and ethnicity. An indicator category was created for missing values found in covariates. Analyses for adherence to individual lifestyle recommendations were mutually adjusted. To examine the pattern of the association, we produced spline curves using 3 knots (25th, 50th and 75th percentiles).

We tested the proportional hazards assumption by Schoenfeld residuals over time and found a moderate deviation from the assumption. Therefore, we additionally evaluated the all-cause mortality association in separate data sets by follow-up time (1-5, 6-10, 11-15, 16-20, or >20 years).

We stratified the all-cause mortality analysis by age, sex, general health, history of cancer, heart disease, or stroke individually and in combination (yes or no to any 1 of these conditions), years since cessation, and CPDs. The stratified analysis by age at smoking initiation was limited to 96 597 participants who provided this information on the 2004-2005 follow-up questionnaire. We performed the likelihood ratio test for heterogeneity by comparing multivariable-adjusted models with and without cross-product terms for the total adherence score and the stratifying variable. Finally, we performed a lag analysis for all-cause mortality excluding deaths that occurred within 2 years of study baseline to investigate potential effects of reverse causality by underlying disease.

Because some former smokers may have restarted smoking during follow-up, we conducted a sensitivity analysis among 91 827 participants who completed the 2004-2005 questionnaire. We also estimated age- and sex-standardized mortality rates stratified by years since cessation and total adherence score categories. All analyses were performed using SAS software, version 9.4 (SAS Institute Inc), and all statistical tests were 2-sided with *P* < .05 considered as statistically significant.

## Results

Our study included 159 937 former smokers (mean [SD] age, 62.6 [5.2] years; 106 912 [66.9%] male; 4442 [2.8%] Black, 2360 [1.5%] Hispanic, 149 742 [93.6%] White, 1844 [1.2%] other race or ethnicity [Asian, Pacific Islander, or American Indian/Alaskan Native], and 1549 [0.9%] unknown race or ethnicity; 64 246 (40.2%) college or postgraduate education). During a mean (SD) follow-up of 18.9 (6.3) years, 86 127 deaths were identified. Former smokers with higher total adherence scores were more likely to be female and older, have more education, and have excellent or very good general health and less likely to have a history of cancer, heart disease, or stroke (Table 2). Former smokers who had higher total adherence score also tended to have smoked fewer CPDs and had quit smoking earlier.

**Table 2.** Demographic, Medical, and Smoking Characteristics by Total Adherence Score to the Lifestyle Recommendations Among Former Smokers

Characteristic	No. (%) of study participants by total adherence score^a^			
	0-2	3-4	5-6	7-8
Total No. (%)	16 558 (10.3)	53 595 (33.5)	62 193 (38.9)	27 591 (17.3)
Sex				
Male	11 944 (72.1)	38 062 (71.0)	40 684 (65.4)	16 222 (58.8)
Female	4614 (27.9)	15 533 (29.0)	21 509 (34.6)	11 369 (41.2)
Age group, y				
<55	2338 (14.1)	6369 (11.9)	6420 (10.3)	2449 (8.9)
55-59	3912 (23.6)	11 331 (21.1)	12 379 (19.9)	5074 (18.4)
60-64	4655 (28.1)	15 428 (28.8)	17 882 (28.7)	7964 (28.9)
65-69	5130 (31.0)	18 458 (34.5)	22 858 (36.8)	10 776 (39.0)
≥70	523 (3.2)	2009 (3.7)	2654 (4.3)	1328 (4.8)
Educational level				
High school or less	4953 (29.9)	13 586 (25.4)	12 690 (20.4)	4648 (16.8)
Post–high school training	1901 (11.5)	5742 (10.7)	6253 (10.1)	2444 (8.9)
Some college	4118 (24.9)	13 601 (25.4)	15 306 (24.6)	6621 (24.0)
College or postgraduate	5148 (31.1)	19 297 (36.0)	26 483 (42.6)	13 318 (48.3)
Unknown	438 (2.6)	1369 (2.4)	1461 (2.4)	560 (2.0)
Race and ethnicity				
Hispanic	254 (1.5)	807 (1.5)	914 (1.5)	385 (1.4)
Non-Hispanic				
Black	577 (3.5)	1640 (3.1)	1666 (2.7)	559 (2.0)
White	15 439 (93.3)	49 976 (93.2)	58 243 (93.6)	26 084 (94.6)
Other^b^	120 (0.7)	627 (1.2)	765 (1.2)	332 (1.2)
Unknown	168 (1.0)	545 (1.0)	605 (1.0)	231 (0.8)
Self-reported health status				
Fair or poor	3938 (23.8)	8097 (15.1)	6143 (9.9)	1564 (5.7)
Good	7007 (42.3)	20 356 (38.0)	19 972 (32.1)	7053 (25.5)
Excellent or very good	5452 (32.9)	24 711 (46.1)	35 578 (57.2)	18 761 (68.0)
Unknown	161 (1.0)	431 (0.8)	500 (0.8)	213 (0.8)
History of cancer	888 (5.4)	2750 (5.1)	2893 (1.8)	1248 (4.5)
History of heart disease^c^	2905 (17.5)	9033 (16.9)	10 617 (17.1)	4572 (16.6)
History of stroke	484 (2.9)	1334 (2.5)	1305 (2.1)	451 (1.6)
Previous smoking patterns				
Years since quitting				
≥10	11 002 (66.5)	38 108 (71.1)	47 406 (76.2)	22 558 (81.8)
5-9	2965 (17.9)	8290 (15.5)	8180 (13.1)	2971 (10.8)
1-4	1839 (11.1)	5128 (9.6)	4585 (7.4)	1449 (5.2)
<1	752 (4.5)	2069 (3.8)	2022 (3.3)	613 (2.2)
Cigarettes smoked per day				
1-10	3030 (18.3)	11 655 (21.8)	16 722 (26.9)	9028 (32.7)
11-20	4281 (25.8)	15 736 (29.4)	20 024 (32.2)	9209 (33.4)
21-30	3449 (20.8)	11 159 (20.8)	12 005 (19.3)	4733 (17.2)
31-40	2742 (16.6)	8003 (14.9)	7536 (12.1)	2736 (9.9)
41-60	2280 (13.8)	5415 (10.1)	4709 (7.6)	1499 (5.4)
>60	776 (4.7)	1627 (3.0)	1197 (1.9)	386 (1.4)

^a^ A sum of adherence scores for the body weight (scores, 0-2), diet (scores, 0-3), alcohol intake (scores, 0-1), and physical activity (scores, 0-2).

^b^ Asian, Pacific Islander, or American Indian/Alaskan Native.

^c^ *P* = .045 (χ^2^ test). *P* < .001 for all other characteristics included in the table.

Former smokers with a higher total adherence score had lower risk of all-cause mortality during follow-up (Table 3). Compared with the lowest score category (0-2), the HRs for all-cause mortality were 0.88 (95% CI, 0.86-0.90) for scores of 3 to 4, 0.80 (95% CI, 0.79-0.82) for scores of 5 to 6, and 0.73 (95% CI, 0.71-0.75) for scores of 7 to 8.^7,8^ On a continuous scale, the risk was 5% lower per 1-score increment (HR per unit increase, 0.95; 95% CI, 0.94-0.95). When excluding deaths that occurred within 2 years of baseline, associations remained similar. Inverse associations were also observed for major causes of death. Compared with the risk in the lowest adherence score category (0-1), HRs in the highest score category^7,8^ were 0.76 (95% CI, 0.72-0.80) for cancer, 0.72 (95% CI, 0.68-0.76) for CVD, and 0.70 (95% CI, 0.64-0.77) for respiratory disease. One adherence score increment was associated with a lower risk by 5% for cancer, 6% for CVD, and 6% for respiratory disease. Spline curves showed additional evidence of a linear association for each mortality outcome (eFigure 1 in the Supplement).

**Table 3.** Hazard Ratios (95% CIs) for All-Cause and Cause-Specific Mortality by Total Adherence Score Among Former Smokers

Mortality	Total adherence score^a^				Continuous^b^
	0-2	3-4	5-6	7-8	
**All causes**					
No. of study participants	16 558	53 595	62 193	27 591	NA
No. of deaths	10 302	30 438	32 365	13 022	NA
Age and sex adjusted	1 [Reference]	0.78 (0.76-0.80)	0.64 (0.63-0.66)	0.53 (0.52-0.54)	0.90 (0.89-0.90)
Age, sex, and general health adjusted^c^	1 [Reference]	0.85 (0.83-0.87)	0.75 (0.73-0.76)	0.65 (0.64-0.67)	0.93 (0.92-0.93)
Age, sex, and smoking adjusted^d^	1 [Reference]	0.81 (0.79-0.83)	0.70 (0.68-0.71)	0.60 (0.59-0.62)	0.92 (0.91-0.92)
Multivariable adjusted^e^	1 [Reference]	0.88 (0.86-0.90)	0.80 (0.79-0.82)	0.73 (0.71-0.75)	0.95 (0.94-0.95)
**All causes excluding death within 2 y**					
No. of study participants	16 248	52 806	61 530	27 383	NA
No. of deaths	10 001	29 679	31 718	12 818	NA
Multivariable adjusted	1 [Reference]	0.88 (0.86-0.90)	0.80 (0.79-0.82)	0.73 (0.71-0.75)	0.95 (0.94-0.95)
**Cancer**					
No. of deaths	2891	8768	9395	3813	NA
Multivariable adjusted	1 [Reference]	0.90 (0.86-0.94)	0.83 (0.79-0.86)	0.76 (0.72-0.80)	0.95 (0.94-0.96)
**CVD**					
No. of deaths	2964	8680	8819	3424	NA
Multivariable adjusted	1 [Reference]	0.89 (0.86-0.93)	0.80 (0.77-0.84)	0.72 (0.68-0.76)	0.94 (0.93-0.95)
**Respiratory disease**					
No. of deaths	971	2702	2506	892	NA
Multivariable adjusted	1 [Reference]	0.92 (0.85-0.99)	0.80 (0.74-0.86)	0.70 (0.64-0.77)	0.94 (0.92-0.95)

Abbreviations: CVD, cardiovascular disease; NA, not applicable.

^a^ A sum of adherence scores for the body weight (scores, 0-2), diet (scores, 0-3), alcohol intake (scores, 0-1), and physical activity (scores, 0-2).

^b^ Adherence score scale in 1-unit increments.

^c^ Perceived general health (excellent or very good, good, fair or poor, or unknown).

^d^ Smoking includes time since quitting (<1, 1-4, 5-9, or ≥10 years or unknown) and smoking intensity (1-10, 11-20, 21-30, 31-40, 41-60, or >60 cigarettes per day or unknown).

^e^ Adjusted for age, sex, race and ethnicity (Hispanic, non-Hispanic Black, non-Hispanic White, other race or ethnicity [Asian, Pacific Islander, or American Indian/Alaskan Native], or unknown), educational level (high school or less, post–high school training, some college, college or graduate school, or unknown), perceived general health (excellent or very good, good, fair or poor, or unknown), time since quitting (<1, 1-4, 5-9, or ≥10 years or unknown), and smoking intensity (1-10, 11-20, 21-30, 31-40, 41-60, or >60 cigarettes per day or unknown).

Adherence to individual recommendations for body weight, diet, physical activity, and alcohol intake was associated with lower risk of all-cause and cause-specific mortality (Table 4). Compared with the lowest score, HRs for all-cause mortality among those who had the highest adherence score were 0.86 (95% CI, 0.84-0.88) for body weight, 0.91 (95% CI, 0.90-0.93) for diet, 0.83 (95% CI, 0.81-0.85) for physical activity, and 0.96 (95% CI, 0.94-0.97) for alcohol intake. Adherence to the body weight recommendation was associated with a lower risk of cancer mortality (HR for highest vs lowest score, 0.93; 95% CI, 0.90-0.97) and CVD (HR, 0.70; 95% CI, 0.68-0.73). However, we observed a nonlinear association for respiratory disease with lower risk for an adherence score of 1 (overweight) (HR, 0.85; 95% CI, 0.80-0.90) and higher risk for an adherence score of 2 (normal) (HR, 1.20; 95% CI, 1.12-1.27) compared with the risk among those with an adherence score of 0 (underweight or obese). Better adherence to dietary recommendations was associated with a lower mortality risk for cancer (HR for highest vs lowest score, 0.84; 95% CI, 0.81-0.87) and respiratory disease (HR, 0.77; 95% CI, 0.72-0.83). Better adherence to the physical activity recommendation was associated with a lower risk for all major causes of death included in the analysis. The HRs for the highest vs lowest adherence score were 0.92 (95% CI, 0.89-0.96) for cancer, 0.80 (95% CI, 0.77-0.83) for CVD, and 0.70 (95% CI, 0.66-0.75) for respiratory disease. Meeting the alcohol intake recommendation was associated with a lower mortality risk for cancer (HR, 0.91; 95% CI, 0.88-0.94) and respiratory disease (HR, 0.92; 95% CI, 0.86-0.97) but not for CVD.

**Table 4.** All-Cause and Cause-Specific Mortality by Adherence Score for the Body Weight, Diet, Alcohol Intake, and Physical Activity Recommendations

Adherence score	No. of study participants	All causes		Cancer		CVD		Respiratory	
		No. of deaths	HR (95% CI)^a^	No. of deaths	HR (95% CI)	No. of deaths	HR (95% CI)	No. of deaths	HR (95% CI)
**Body weight^b^**									
0	39 511	23 657	1 [Reference]	6256	1 [Reference]	7342	1 [Reference]	1983	1 [Reference]
1	70 400	37 666	0.85 (0.83-0.86)	11 331	0.94 (0.91-0.97)	10 510	0.76 (0.74-0.79)	2695	0.85 (0.80-0.90)
2	50 026	24 804	0.86 (0.84-0.88)	7280	0.93 (0.90-0.97)	6035	0.70 (0.68-0.73)	2393	1.20 (1.12-1.27)
**Diet^c^**									
0	39 984	22 695	1 [Reference]	6824	1 [Reference]	6133	1 [Reference]	2140	1 [Reference]
1	39 982	21 826	0.98 (0.96-1.00)	6403	0.95 (0.92-0.98)	5936	1.00 (0.97-1.04)	1817	0.93 (0.87-0.99)
2	39 985	21 036	0.94 (0.92-0.96)	5937	0.88 (0.85-0.91)	5920	1.01 (0.98-1.05)	1680	0.89 (0.84-0.95)
3	39 986	20 570	0.91 (0.90-0.93)	5703	0.84 (0.81-0.87)	5898	1.03 (0.99-1.07)	1434	0.77 (0.72-0.83)
**Physical activity^d^**									
0	22 521	14 148	1 [Reference]	3657	1 [Reference]	4229	1 [Reference]	1465	1 [Reference]
1	56 604	30 612	0.88 (0.86-0.90)	8807	0.95 (0.91-0.99)	8534	0.86 (0.82-0.89)	2634	0.83 (0.78-0.89)
2	80 812	41 367	0.83 (0.81-0.85)	12 403	0.92 (0.89-0.96)	11 124	0.80 (0.77-0.83)	2972	0.70 (0.66-0.75)
**Alcohol intake^e^**									
0	27 889	15 166	1 [Reference]	4734	1 [Reference]	3886	1 [Reference]	1290	1 [Reference]
1	132 048	70 961	0.96 (0.94-0.97)	20 133	0.91 (0.88-0.94)	20 001	1.01 (0.98-1.05)	5781	0.92 (0.86-0.97)

Abbreviations: CVD, cardiovascular disease; HR, hazard ratio.

^a^ Adjusted for age, sex, race, and ethnicity (Hispanic, non-Hispanic Black, non-Hispanic White, other race or ethnicity [Asian, Pacific Islander, or American Indian/Alaskan Native], or unknown), educational level (high school or less, post–high school training, some college, college or graduate school, or unknown), perceived general health (excellent or very good, good, fair or poor, or unknown), time since quitting (<1, 1-4, 5-9, or ≥10 years or unknown), and smoking intensity (1-10, 11-20, 21-30, 31-40, 41-60, or >60 cigarettes per day or unknown), and individual lifestyle recommendations were mutually adjusted.

^b^ The World Health Organization’s classifications by body mass index (calculated as weight in kilograms divided by height in meters squared): obese or underweight (≥30 or <18.5) (score, 0), overweight (25 to <30) (score, 1), and normal (18.5 to <25) (score, 2).

^c^ Healthy Eating Index 2015 total score quartiles.

^d^ Adherence to the *Physical Activity Guidelines for Americans*, second edition^23^ based on reported time of moderate and vigorous physical activity: never or rarely (score, 0), less than 1 to 3 hours per week (score, 1), and 4 or more hours per week (score, 2).

^e^ Adherence to the alcohol consumption recommendation by the *Dietary Guidelines for Americans, 2020-2025*.^25^ Women who reported drinking 1 or fewer alcoholic drinks per day and men who reported drinking 2 or fewer alcoholic drinks per day were scored as adherent (score, 1). Participants who reported drinking more than these amounts received an adherence score of 0.

We found that all-cause mortality risk was lower with a higher total adherence score, regardless of potential effect modifiers (eFigure 2 in the Supplement). The association was stronger among participants with very good or excellent general health (HR for highest vs lowest adherence score category, 0.69; 95% CI, 0.66-0.72) compared with participants with poor or fair general health (HR, 0.79; 95% CI, 0.74-0.85) (*P* = .02 for heterogeneity) and those without comorbid conditions (cancer, CVD, or stroke) (HR, 0.69; 95% CI, 0.66-0.71) compared with those with comorbid conditions (HR, 0.75; 95% CI, 0.71-0.79). However, inverse associations were observed regardless of health status and comorbid conditions. Higher total adherence was associated with lower all-cause mortality regardless of years since cessation at baseline, CPDs, or age at smoking initiation.

The association with all-cause mortality was stronger with shorter follow-up time (ie, earlier in follow-up) than longer follow-up time (ie, later in follow-up) (eFigure 2 in the Supplement). The HRs for the highest vs lowest adherence score categories were 0.66 (95% CI, 0.60-0.73) for 1 to 5 years of follow-up, 0.67 (95% CI, 0.63-0.72) for 6 to 10 years of follow-up, 0.71 (95% CI, 0.67-0.75) for 11 to 15 years of follow-up, 0.74 (95% CI, 0.71-0.78) for 16 to 20 years of follow-up, and 0.77 (95% CI, 0.73-0.81) for more than 20 years of follow-up.

Former smokers who had quit earlier had lower mortality rates than those who quit more recently (Figure). In addition, the mortality rates for participants with the highest adherence scores were approximately half of the mortality rates for participants with the lowest adherence scores. This difference persisted across categories of years since cessation. Among participants who quit smoking 10 years or more before baseline, mortality rates were 3321 per 100 000 person-years for adherence scores of 0 to 2, 2595 per 100 000 person-years for adherence scores of 3 to 4, 2152 per 100 000 person-years for adherence scores of 5 to 6, and 1806 per 100 000 person-years for adherence scores of 7 to 8.

**Figure.**
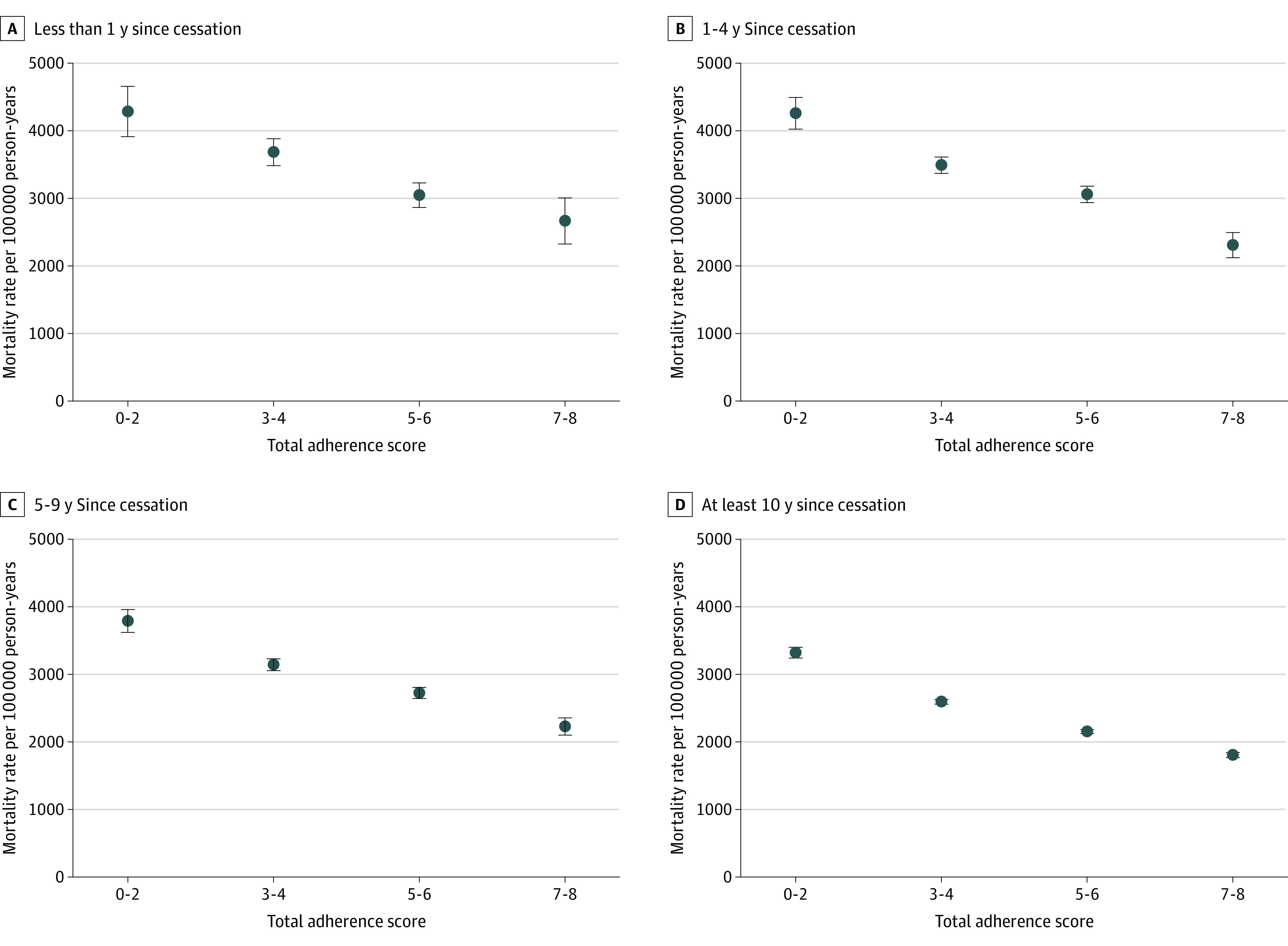
Age- and Sex-Adjusted Mortality Rate by Total Adherence Score Stratified by Years Since Cessation Among Former Smokers Total adherence score is a sum of adherence scores for the body weight (0-2), diet (0-3), alcohol intake (0-1), and physical activity (0-2), ranging from 0 to 8. Error bars indicate 95% CIs.

In a sensitivity analysis among the subset of participants (n = 91 827) who completed the later 2004-2005 follow-up questionnaire, 95% remained categorized as former smokers. Similar inverse associations were observed between a total adherence score and all-cause mortality regardless of whether they restarted smoking after study baseline (HR for highest vs lowest category, 0.66; 95% CI, 0.64-0.69 for nonsmoking and HR, 0.53; 95% CI, 0.38-0.72 for smoking in the follow-up) as was observed overall (eTable 2 in the Supplement).

## Discussion

In this study of 159 937 older US adults who were former smokers, better adherence to evidence-based recommendations for body weight, diet, physical activity, and alcohol intake was associated with a lower risk of mortality from all causes, cancer, CVD, and respiratory disease. Associations were observed regardless of health status, comorbid conditions, number of CPDs, years since cessation, and age at smoking initiation.

In the general population, many studies^15,16,17,19,26^ have found that better adherence to evidence-based healthy lifestyle guidelines is associated with lower disease and mortality risk. In a meta-analysis^15^ of 3 large cohort studies in the US and Europe, a 1-unit increment in adherence to the 2007 WCRF/AICR recommendations for body weight, diet, and alcohol intake was associated with a 10% lower mortality risk and with lower risk of breast, colorectal, and lung cancer. In another meta-analysis^16^ of more than 360 000 adults in the US and Europe, a 1-point increment in adherence to the WCRF/AICR recommendations was associated with a 6% lower risk of all cancer and lower risk of cancers of the colorectum (by 16%) and prostate (by 6%). Similarly, individuals who were in the highest quintile of adherence to the ACS guidelines for body weight, diet, alcohol intake, physical activity, and smoking had a 37% lower risk of all-cause mortality and a 24% lower risk of incident breast cancer compared with individuals in the lowest quintile.^19^

However, few studies have been conducted among former smokers. Most prior studies^10,15,17,19,26^ included quitting smoking as one of the recommendations or adjusted the analysis for smoking. In the Nurse’s Health Study and the Health Professionals Follow-up Study, associations of body weight, physical activity, and alcohol intake with incident cancer were examined separately among current smokers and nonsmokers but not among former smokers specifically.^27^ Adherence to the ACS guidelines for body weight, physical activity, diet, and alcohol intake was associated with a lower risk of cancer mortality across the strata of smoking status in a prior analysis^28^ in the NIH-AARP cohort. In the current study, we expanded this previous work^28^ to include an additional 4 years of follow-up and by examining all-cause and cause-specific mortality. Furthermore, we focused on former smokers who have unique health risks and carefully examined whether associations varied by prior smoking pattern.

### Strengths and Limitations

The current study has several strengths. Detailed data about lifestyle enabled us to evaluate participants’ adherence to important evidence-based lifestyle recommendations. With a large sample size and long follow-up, we were able to assess mortality risk by major causes of death and perform stratified analyses to examine potential effect modifiers. Smoking history was assessed in detail, and thus we were able to adjust and stratify our analyses by lifetime smoking patterns.

This study also had some limitations. As an observational study, residual and unmeasured confounding is of concern, especially by smoking patterns and underlying health conditions. Participants adhering more to lifestyle recommendations tended to quit somewhat earlier and had smoked somewhat less than participants who adhered less. Former smokers may have contracted smoking-related diseases or had poorer overall health and thus quit. In addition, NIH-AARP participants with poorer health may be less physically active than participants with better health. However, we observed robust associations after comprehensive adjustment for potential confounders. We also found similar inverse associations in analyses stratified by prior CPDs, years since cessation, age at smoking initiation, comorbid conditions, and general health and after excluding deaths occurring within 2 years of baseline. Smoking and other aspects of lifestyle were self-reported based on participants’ recall and assessed once at study baseline, which could have led to misclassification and measurement error. Changes in lifestyle during follow-up could have occurred, which is consistent with our finding of weaker associations for deaths occurring after more years of follow-up. Potentially of most concern, some former smokers could have restarted smoking during follow-up. However, among the subset of 91 827 former smokers who additionally completed a questionnaire in 2004 to 2005, 95% remained categorized as former smokers, and we found the robust inverse associations regardless of smoking status in the follow-up survey. In addition, participants were predominantly White individuals and had relatively higher socioeconomic status than the US general population.^20^ Future studies should be performed in more diverse populations.

## Conclusions

The benefits of smoking cessation are strong and clear. However, even after cessation, former smokers have higher disease and mortality risks than never smokers. In this cohort study of 159 937 former smokers, better adherence to evidence-based recommendations for body weight, diet, physical activity, and alcohol intake was associated with lower mortality from all causes and major causes of death, regardless of prior smoking patterns. These results provide evidence that former smokers benefit from adhering to lifestyle recommendations, as do other groups.

## Notes

Supplement. **eTable 1.** Individual Lifestyle Recommendation Adherence Score and Total Adherence Score Among Former Smokers in the NIH-AARP Diet and Health Study **eFigure 1.** All-Cause and Cause-Specific Mortality Risk by Total Recommendation Adherence Score in a Continuous Scale **eFigure 2.** All-Cause Mortality Among Former Smokers in the Highest Total Adherence Score Category (7-8) Relative to the Lowest Score Category (0-2) Stratified by Potential Effect Modifiers **eTable 2.** All-Cause Mortality Risk by Total Adherence Score Stratified by Smoking Status Reported in the 2004-2005 Follow-up Questionnaire
