# Supplementary material for: Association of Adherence to Healthy Lifestyle Recommendations With All-Cause and Cause-Specific Mortality Among Former Smokers
Source: JAMA Netw Open. 2022 Sep 22;5(9):e2232778. doi: 10.1001/jamanetworkopen.2022.32778 (PMC9500560; doi:10.1001/jamanetworkopen.2022.32778)
Supplement: Supplement. — eTable 1. Individual Lifestyle Recommendation Adherence Score and Total Adherence Score Among Former Smokers in the NIH-AARP Diet and Health Study eFigure 1. All-Cause and Cause-Specific Mortality Risk by Total Recommendation Adherence Score in a Continuous Scale eFigure 2. All-Cause Mortality Among Former Smokers in the Highest Total Adherence Score Category (7-8) Relative to the Lowest Score Category (0-2) Stratified by Potential Effect Modifiers eTable 2. All-Cause Mortality Risk by Total Adherence Score Stratified by Smoking Status Reported in the 2004-2005 Follow-up Questionnaire [file jamanetwopen-e2232778-s001.pdf]

Supplementary Online Content

Inoue-Choi M, Ramirez Y, Fukunaga A, Matthews CE, Freedman ND. Association of adherence to healthy lifestyle recommendations with all-cause and cause-specific mortality among former smokers. *JAMA Netw Open*. 2022;5(9):e2232778. doi:10.1001/jamanetworkopen.2022.32778

eTable 1. Individual Lifestyle Recommendation Adherence Score and Total Adherence Score Among Former Smokers in the NIH-AARP Diet and Health Study

eFigure 1. All-Cause and Cause-Specific Mortality Risk by Total Recommendation Adherence Score in a Continuous Scale

eFigure 2. All-Cause Mortality Among Former Smokers in the Highest Total Adherence Score Category (7-8) Relative to the Lowest Score Category (0-2) Stratified by Potential Effect Modifiers

eTable 2. All-Cause Mortality Risk by Total Adherence Score Stratified by Smoking Status Reported in the 2004-2005 Follow-up Questionnaire

This supplementary material has been provided by the authors to give readers additional information about their work.

© 2022 Inoue-Choi M et al. *JAMA Network Open.*

eTable 1. Individual lifestyle recommendation adherence score and total adherence score among former smokers in the NIH-AARP Diet and Health Study

	**No. (%) of study participants by total Adherence Score[a]**			
	**0-2**	**3-4**	**5-6**	**7-8**
Total No. (%)	16,558 (10.3)	53,595 (33.5)	62,193 (38.9)	27,591 (17.3)
Body weight[b,c]				
0	11,619 (70.2)	18,544 (34.6)	9,348 (15.0)	0
1	4,626 (27.9)	26,911 (50.2)	30,8107 (49.6)	8,056 (29.2)
2	313 (1.9)	8,140 (15.2)	22,038 (35.4)	19,535 (70.8)
Diet[c,d]				
0	12,989 (78.5)	22,753 (42.4)	4,242 (6.8)	0
1	3,362 (20.3)	19,925 (37.2)	16,695 (26.9)	0
2	207 (1.2)	9,068 (16.9)	24,637 (39.6)	6,073 (22.0)
3	0	1,849 (3.5)	16,619 (26.7)	21,518 (78.0)
Physical activity[c,e]				
0	9,779 (59.1)	9,704 (18.1)	3,038 (4.9)	0
1	6,325 (38.2)	25,940 (48.4)	21,083 (33.9)	3,256 (11.8)
2	454 (2.7)	17,951 (33.5)	38,072 (61.2)	24,335 (88.2)
Alcohol intake[c,f]				
0	4,629 (28.0)	10,484 (19.6)	10,481 (16.9)	2,295 (8.3)
1	11,929 (72.0)	43,111 (80.4)	51,712 (83.1)	25,296 (91.7)

[a] A sum of adherence scores for the body weight (scores, 0-2), diet (scores, 0-3), alcohol intake (scores, 0-1), and physical activity (scores, 0-2)

[b] 0: Body mass index (BMI; kirograms per meters squared) < 18.5 or ≥ 30; 1: 25 to < 30; and 2: 18.5 to < 25

[c] *P* <.001

[d] Healthy Eating Index-2015 total score; 0: quatile 1 (Q1), and 1: Q2, 2: Q3, 3: Q4

[e] Adherence to the *Physical Activity Guidelines for Americans, Second Edition* based on reported time of moderate and vigorous physical activity; 0: no adherence (never or rarely); 1: partial adherence (less than 1 to 3 hours per week); and 2: adherence (4 hours per week or more).

[f] Adherence to the alcohol consumption recommendation by the *Dietary Guidelines for Americans, 2020-2025*; 0: no adherence (more than 2 alcoholic drinks per day for men and more than 1 alcoholic drink per day for women; and 1: adherence (2 or fewer alcoholic drinks per day for men and 1 or fewer alcoholic drinks per day for women.

© 2022 Inoue-Choi M et al. *JAMA Network Open.*

eFigure 1. All-cause and cause-specific mortality risk by total recommendation adherence score in a continuous scale

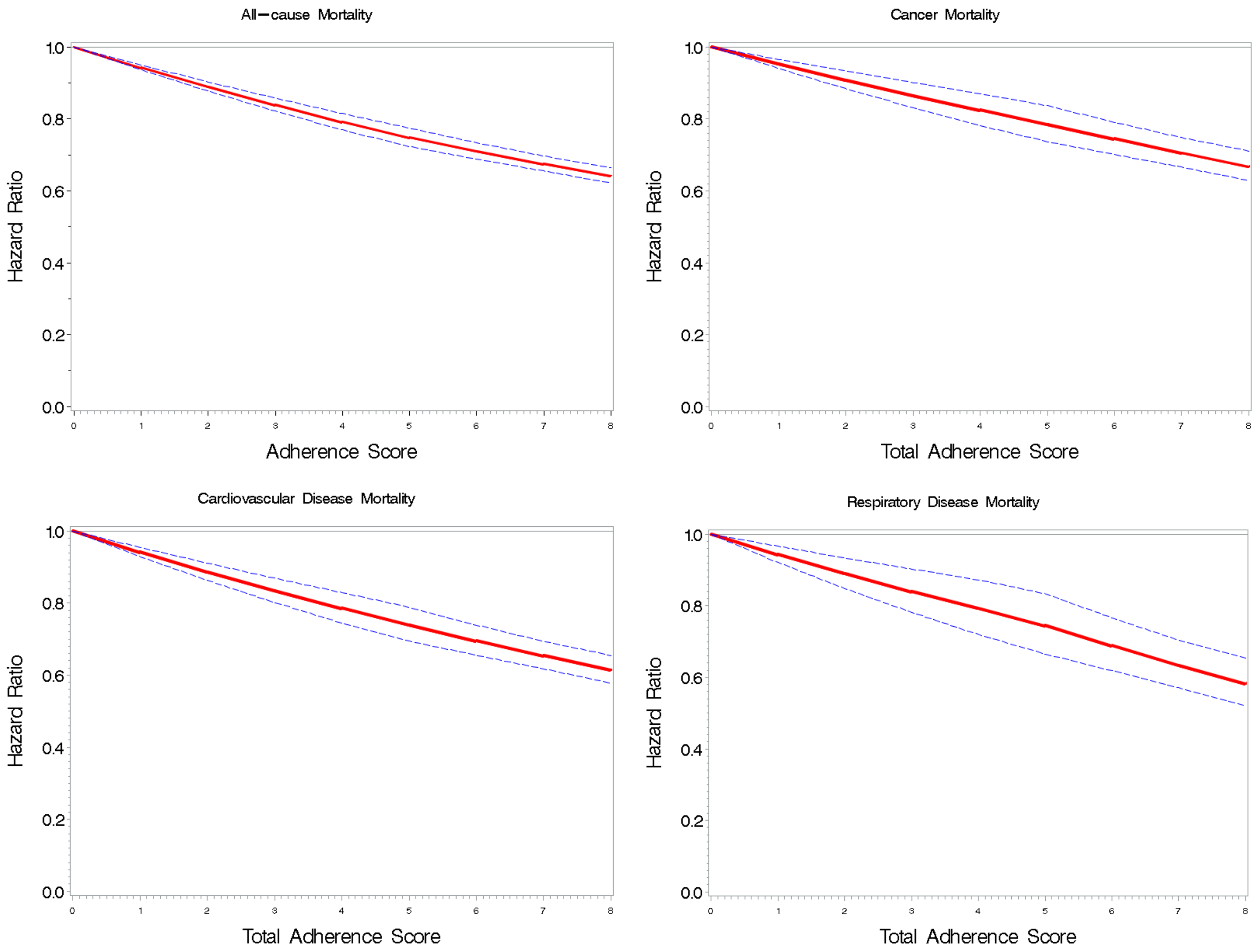

© 2022 Inoue-Choi M et al. *JAMA Network Open.*

Solid lines represent hazard ratios and dotted lines resprent 95% CIs.

Hazard ratios were adjusted for age, sex, race or ethnicity (Hispanic, non-Hispanic black, non-Hispanic white, other, or unknown), educational level (high school or less, post high school training, some college, college or graduate school, or unknown), perceived general health (excellent or very good, good, fair or poor, or unknown), time since quitting (<1, 1-4, 5-9, or ≥10 years, or unknown), and smoking intensity (1-10, 11-20, 21-30, 31-40, 41-60, or >60 cigarettes per day, or unknown).

© 2022 Inoue-Choi M et al. *JAMA Network Open.*

eFigure 2. All-cause mortality among former smokers in the highest total adherence score category (7-8) relative to the lowest score category (0-2) stratified by potential effect modifiers

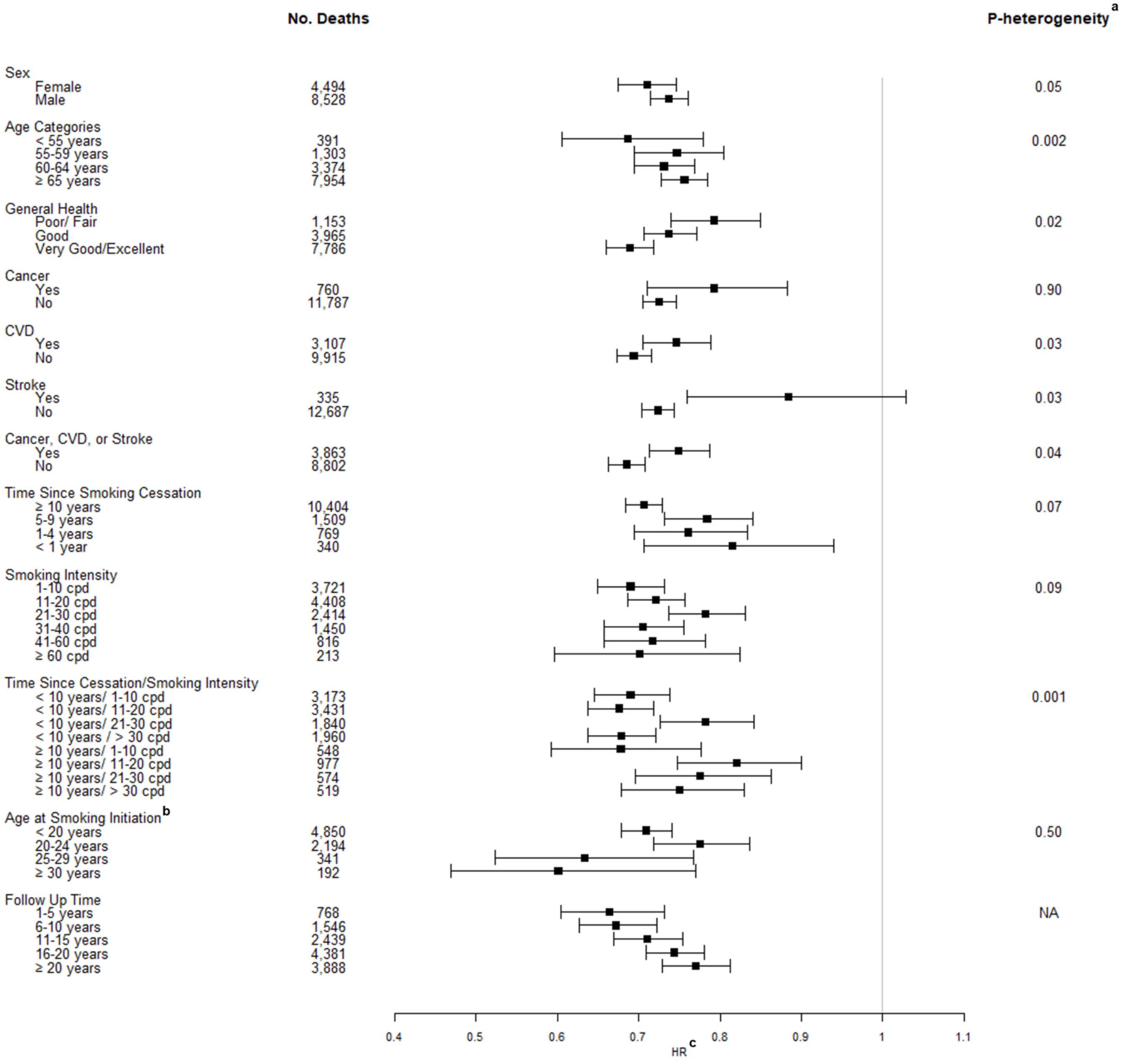

Abbribiations: CVD, cardiovascular disease; HR, hazard ratio

[a] *P* for heterogeneity from the likelihood tests comparing the multivariable-adjusted models with and without the cross-product terms of total adherence score category and each stratifying factor

[b] The analysis was limited to 96,597 participants who reported this information on the 2004-2005 follow-up questionnaire.

[c] Hazard ratios and 95% CIs were adjusted for age, sex, race or ethnicity (Hispanic, non-Hispanic black, non-Hispanic white, other, or unknown), educational level (high school or less, post high school training, some college, college or graduate school, or unknown), perceived general health (excellent or very good, good, fair or poor, or unknown), time since quitting (<1, 1-4, 5-9, or ≥10 years, or unknown), and smoking intensity (1-10, 11-20, 21-30, 31-40, 41-60, or >60 cigarettes per day, or unknown).

© 2022 Inoue-Choi M et al. *JAMA Network Open.*

eTable 2. All-cause mortality risk by total adherence score stratified by smoking status reported in the 2004-2005 follow-up questionnaire

	**Total adherence score**[a]				**Continuous**[b]
	**0 - 2**	**3 - 4**	**5 - 6**	**7 - 8**	
Non-smoking					
No. of study participants	7,972	27,991	34,472	16,302	NA
No. of deaths	4,287	13,928	15,680	6,732	NA
HR (95% CI)[c]	1.00 (ref)	0.86 (0.83-0.89)	0.76 (0.73-0.78)	0.66 (0.64-0.69)	0.93 (0.93-0.94)
Smoking					
No. of study participants	172	593	493	170	NA
No. of deaths	111	339	255	64	NA
HR (95% CI)	1.00 (ref)	0.81 (0.65-1.00)	0.75 (0.60-1.94)	0.53 (0.38-0.72)	0.84 (0.77-0.92)

Abbribiations: HR, hazard ratio; NA: not applicable.

[a] A sum of adherence scores for the body weight (scores, 0-2), diet (scores, 0-3), alcohol intake (scores, 0-1), and physical activity (scores, 0-2)

[b] Adherence score scale in 1-unit increments

[c] Adjusted for age, sex, race or ethnicity (Hispanic, non-Hispanic black, non-Hispanic white, other, or unknown), educational level (high school or less, post high school training, some college, college or graduate school, or unknown), perceived general health (excellent or very good, good, fair or poor, or unknown), and smoking intensity reported on the 2004-2005 follow-up questionnaire (≤10, 11-20, 21-30, 31-40, 41-60, or >60 cigarettes per day, or unknown).

© 2022 Inoue-Choi M et al. *JAMA Network Open.*
